# The Application of CRISPR/Cas9 Technology for Cancer Immunotherapy: Current Status and Problems

**DOI:** 10.3389/fonc.2021.704999

**Published:** 2022-01-17

**Authors:** Luyao Wang, Yurong Chen, Xinrui Liu, Ziyi Li, Xiangpeng Dai

**Affiliations:** ^1^ Key Laboratory of Organ Regeneration and Transplantation of Ministry of Education, First Hospital, Jilin University, Changchun, China; ^2^ National-Local Joint Engineering Laboratory of Animal Models for Human Disease, First Hospital, Jilin University, Changchun, China; ^3^ Neurosurgery Department, First Hospital, Jilin University, Changchun, China

**Keywords:** cancer, CRISPR/Cas9, immunotherapy, oncolytic viruses, immune checkpoints inhibition, CAR-T therapy

## Abstract

Cancer is one of the main causes of disease-related deaths in the world. Although cancer treatment strategies have been improved in recent years, the survival time of cancer patients is still far from satisfied. Cancer immunotherapy, such as Oncolytic virotherapy, Immune checkpoints inhibition, Chimeric antigen receptor T (CAR-T) cell therapy, Chimeric antigen receptor natural killer (CAR-NK) cell therapy and macrophages genomic modification, has emerged as an effective therapeutic strategy for different kinds of cancer. However, many patients do not respond to the cancer immunotherapy which warrants further investigation to optimize this strategy. The clustered regularly interspaced short palindromic repeats and CRISPR-associated protein 9 (CRISPR/Cas9), as a versatile genome engineering tool, has become popular in the biology research field and it was also applied to optimize tumor immunotherapy. Moreover, CRISPR-based high-throughput screening can be used in the study of immunomodulatory drug resistance mechanism. In this review, we summarized the development as well as the application of CRISPR/Cas9 technology in the cancer immunotherapy and discussed the potential problems that may be caused by this combination.

## Introduction

Given that the incidence of cancer is gradually increasing even with the improved prognosis techniques (1), methods for cancer therapy are widely investigated and the chemotherapy, radiotherapy and surgery are commonly used to prolong survival time of cancer patients. However, the side effects and toxicity of different treatments frequently emerged and subsequently reduced patients’ life quality and even led to death (2). In recent years, immunotherapy has provided novel direction for cancer therapy (3). Oncolytic virus therapy, chimeric antigen receptor T therapy, immune checkpoints blockade and genetically engineered macrophages have provided multi-mode methods to target and destroy cancer cells. However, there are still some associated problems in tumor immunotherapy which limited its wide application. Somatic mutations may cause resistance to tumor immunotherapy and reduce the efficiency of immunotherapy (4). Although oncolytic virus therapy has been proved to be effective in cancer treatment (5–7), the main methods of adenovirus genome engineering are time-consuming, multi-step and inefficient. Immune checkpoint blockade therapy has raised attention with the development of immunology. CAR-T cell therapy has shown clinical effect in multiple types of tumors, especially hematological tumors (8). However, it is difficult to obtain enough qualified T cells from cancer patients and to transfer T cells from healthy donors to patients without causing problems. Therefore, developing a strategy to produce universal or off-the-shelf CAR-T cells has raised attention of researchers. The endogenous αβ T-cell receptor (TCR) and the human leukocyte antigen (HLA) molecules on allogeneic T cells are responsible for safety problems of allogeneic CAR-T cell transfer.

Clustered regularly interspaced short palindromic repeats (CRISPR)-associated 9 system, as a versatile genome engineering tool, has become popular in the researches of biology and genetic therapy because of its ability to edit genomes of various organisms efficiently (9–11). The important step of CRISPR/Cas9 is that the single guide RNA (sgRNA) directs the DNA endonuclease Cas9 to specific DNA sequences to create double-strand DNA breaks site-specifically (12). The first step is the recognition of the protospacer adjacent motif (PAM) by Cas9. Then the combination of sgRNA and the PAM site attracts Cas9 to generate a target-specific double-strand break (DSB), which rapidly stimulates one of the two DNA repair pathways: homology-directed repair (HDR), or non-homologous end-joining (NHEJ). The NHEJ repair leads to the direct ligation of the cleaved strands producing insertions–deletions (InDels), which is commonly used for gene disruption. The HDR follows a directional correction strategy in which an exogenous repair template with the desired nucleotide sequence mediates the process. Compared with previous genomic editing techniques including zinc-finger nucleases (ZFNs) and transcription activator-like effector nucleases (TALENs), the CRISPR/Cas9 technology is simpler, preciser and more operational (13). CRISPR/Cas9 system is considered as a promising candidate for optimizing cancer immunotherapy since it has powerful gene editing efficiency. High throughput screening based on CRISPR can be used to identify novel drug targets, biomarkers and drug resistance related genes in cancer immunotherapy (14). CRISPR technology may provide a more convenient method to engineer the adenovirus genome. Using CRISPR/Cas9 technology to knock out immune checkpoints such as programmed death-1 (PD-1), programmed cell death ligand 1 (PD-L1) and cytotoxic T-lymphocyte antigen 4 (CTLA-4) may provide a new direction for cancer immunotherapy (15). CRISPR/Cas9 technology also can be used to enhance anti-tumor effect of CAR-T cells by optimizing the manufacture of CAR-T cells and produce allogeneic CAR-T cells without graft-versus-host disease (GVHD) by disrupting T-cell receptor (TCR) beta chain and beta-2-microglobulin (B2M, an essential subunit of the HLA-I) (16, 17). Furthermore, CRISPR/Cas9 could improve the phagocytosis of macrophages to tumor cells by knocking out signal regulatory protein-α (SIRP-α) (18). In this review, we will discuss the application of CRISPR technology in cancer immunotherapy (**Supplemental Table 1**).

## The Development of Cancer Immunotherapy

**Figure 1:**
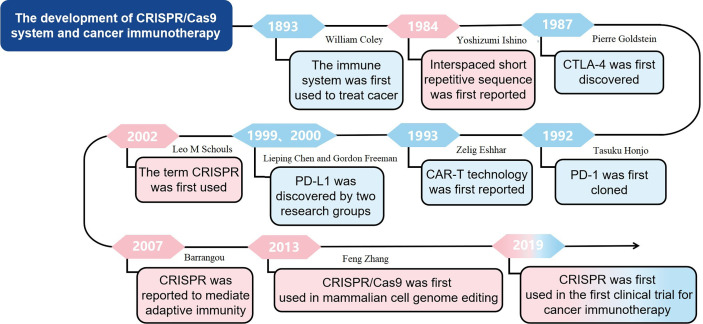
The development of CRISPR/Cas9 system and cancer immunotherapy.

The earliest use of immune system to treat cancer can be traced back to 1893 when William Coley used living bacteria as an immune stimulant. However, the clinical efficacy was limited because tumor cells have the ability to avoid being recognized and eliminated by immune system (19). Recently, with the in-depth understanding of anti-tumor immunity, tumor immune escape mechanism and new targets of immunotherapy, tumor immunotherapy has gradually become important for cancer treatment. The immunocheckpoint inhibitors targeting cytotoxic T-lymphocyte antigen 4 and programmed death-1 (20, 21), and CAR-T cell therapy have achieved clinical success (22). CTLA-4 was originally discovered in 1987 by Pierre Goldstein (23). PD-1 was first cloned in 1992 by Tasuku Honjo, while PD-L1, which is the ligand for PD-1, was discovered independently by two research groups led by Lieping Chen in 1999 and Gordon Freeman in 2000 (24, 25). In 1993, Zelig Eshhar first reported CAR technology (26). In this research, Zelig Eshhar et al. constructed chimeric genes containing a single-chain Fv domain (scFv) of an antibody, which could be expressed as functional surface receptors and provide T cells and other effector lymphocytes with antibody-type recognition directly coupled to cellular activation. The development of cancer immunotherapy, especially immune checkpoint inhibition and CAR-T cell therapy, marks the beginning of a new era of cancer therapy. However, immunotherapy is only effective for a subset of cancers and a fraction of patients (27). The underlying mechanisms of CRISPR technology in tumor immunotherapy warrant further in-deep investigation. In general, the application of CRISPR technology in tumor immunotherapy may improve the effect of therapy and expand the application scope of tumor immunotherapy. The development of cancer immunotherapy was shown in **Figure 1**.

## The Development of CRISPR/Cas9 Technology

In 1987, Yoshizumi Ishino reported a set of interspaced short repetitive sequences downstream of the Escherichia coli iap gene, which is the first report of CRISPR (28). However, at that time, the function of these sequences was not fully studied. During the next 15 years, such repetitive sequences were also found in other bacteria and archaea (29). Until the year of 2002, the term CRISPR was first used to describe such sequences (30) and in 2005, Bolotin reported that CRISPR functions as the immune machinery against foreign DNA invasion (31). Given that the CRISPR-associated (Cas) genes always located near the CRISPR loci, there might be a potential functional relationship between Cas genes and CRISPR loci (31). Interestingly, in 2007, Barrangou reported a type II CRISPR-Cas system as an adaptive defensive system against phage infection (32). In 2013, the CRISPR/Cas9 was firstly used to edit mammalian cell genome, which is a great progress in application of CRISPR/Cas9 technology (33). The development of CRISPR/Cas9 system was shown in **Figure 1**.

According to endonuclease characteristics, CRISPR/Cas system has been divided into two main classes and six main types. The class I system includes type I, III and IV, while the class II system contains type II, V and VI (34). It is well known that the Cas9 nuclease belongs to type II system and class 2 (35). The Cas9 nuclease contains RuyC and HNH as two catalytic active domains and functions as molecular scissors for the DNA strands cutting (35). Compared with other gene editing tools, such as transcription activator-like effector nucleases (TALENs) and zinc-finger nucleases (ZFNs), instead of synthesizing a cumbersome guiding protein, CRISPR only needs a sgRNA to target a new DNA sequence, which makes gene editing easier (35). Moreover, CRISPR/Cas system allows multiple sgRNAs to edit several sites simultaneously which promotes the editing efficiency (33).

Due to the advantages of simplicity and high success rate, CRISPR technology was developed rapidly in the field of gene editing and its application was expanded to functional genomics, cancer researches and gene therapy researches (36).

## The Application of CRISPR Technology in the Identification of Key Genes for Cancer Immunotherapy

In most cancers, gene aberrations are the products of neoplastic evolution (37). Somatic mutations can contribute to the production of neoantigens eliciting potent T cell responses in current immunotherapies (4). However, somatic mutations includes mutations resistant to anti-tumour response in immunotherapy. For example, it has been reported that acquired resistance to PD-1 blockade therapy in patients was related to the loss-of-function mutations of beta-2-microglobulin (B2M), Janus kinases 1 (JAK1) and Janus kinase 2 (JAK2) (38). Therefore, CRISPR-based high-throughput screening has been used to identify novel drug targets, biomarkers and drug resistance related genes (14).

The CRISPR/Cas9 screening has been applied to cancer immunotherapy. To identify the essential genes in tumor cells for the ‘effector function of T cells’ (EFT), researchers developed a ‘two cell-type’ (2CT) CRISPR assay which consists human T cells as effectors and melanoma cells as targets (39). Researchers used a genome-scale CRISPR/Cas9 library containing around 123,000 single-guide RNAs to profile genes whose loss in tumour cells damaged the effector function of CD8+ T cells and contributed to the T-cell-based immunotherapy resistance (14). Ultimately, they found several previously undescribed genes and microRNAs which play a role in promoting T cell damage to tumor (14). There is also one study using genome-wide CRISPR/Cas9 screen to explore the mechanism underlying immunomodulatory drugs (IMiDs) sensitivity (40). Furthermore, researchers used CRISPR screening to study the mechanism of T-cell activation and figured out *FAM49B* as a novel target of tumor immune drugs (41). Therefore, the application of CRISPR/Cas9 screening in the study of immunomodulatory drug resistance mechanism is of great significance for improving drug sensitivity and overcoming drug resistance.

## The Application of CRISPR/Cas9 in Oncolytic Virotherapy

Oncolytic viruses (OVs) are tumor-killing viruses which can selectively infect and kill cancer cells without damage to normal tissues (42). They can produce and release new virus progeny in the infected cancer cells, and induce anti-tumor immune responses that specifically destroy uninfected cancer cells. Therefore, oncolytic virotherapy provides a multi-mode method to target and kill cancer cells specifically and effectively (43).

Clinical and preclinical trials have shown that OVs are effective in the cancer treatment (5–7). In one phase II trial, direct intratumoral injection of talimogene laherparepvec (T-VEC), a second-generation oncolytic herpesvirus coding for granulocyte-macrophage colony-stimulating factor (GM-CSF), to 50 patients with unresectable metastatic melanoma achieved 26% response rate (5).

CRISPR/Cas9 technology can be used to better manipulate the genome of viruses. The homologous recombination system based on bacteria (44), the bacterial artificial chromosome (BAC) system (45) and the hybrid yeast-bacteria cloning system (46) are the three main methods to engineer adenovirus genome. However, these methods are laborious, multi-step and inefficient. In recent years, CRISPR technology has been successfully applied to manipulate the genome of various viruses, including vaccinia virus (VV), herpes simplex virus (HSV) and adenoviral vector (ADV) (47–49). For example, the CRISPR/Cas9 system can introduce *dsRed* gene into ADV genome with efficiency of 2%-3% without off-target mutations (47–49). CRISPR technology has also been successfully used to generate mutations efficiently in the HSV-1 target region (48). The combination of gRNA-guided Cas9 and a homologous repair donor DNA has been used to construct the mutant *HSV-1* expressing *EGFP* reporter gene. The efficiency of homologous recombination is just 0.0000015% of the total plaques using the control cells (not transfected with gRNA-guided Cas9), while the efficiency of homologous recombination is improved to 8.41% of total plaques using cells transfected with gRNA-guided Cas9 and a repair donor DNA (50). Using CRISPR/Cas9 to engineer adenovirus genome is more efficient than traditional methods.

CRISPR/Cas9-mediated genome editing improved tumor selectivity and enhance immune stimulation through engineering oncolytic viruses. It has been reported that the *E1B* gene encoding 55-kilodalton (kDa) protein binds to and inactivates p53 (51). The mutant adenovirus which does not express the 55-kilodalton E1B protein can replicate and lyse p53 deficient tumor cells but not cells with functional p53 (51). E1B gene-attenuated adenovirus ONYX-015 has been reported to cause tumor-specific cytolysis and antitumor efficacy (52). Deletion of the thymidine kinase (TK) region in the virus genome is one of the most common modifications to improve tumor selectivity. In one study, the CRISPR/Cas9 system was applied to replace the *TK* gene by the *RFP* gene effectively, and subsequently enhanced the selectivity of oncolytic viruses to tumor (49). Therefore, the CRISPR/Cas9 system may play an important role in further development of oncolytic viruses, thus providing opportunities for the progress of oncolytic virotherapy.

## The Application of CRISPR/Cas9 in Immune Checkpoints Blockade Therapy

Although the immune system plays a significant role in cancer control, cancer cells can still escape the host immune surveillance. The acquisition of this immune tolerance is an important reason for the growth and development of cancer, and may be also one of the reasons for the resistance to traditional cancer immunotherapy. Therefore, attention has been paid to develop therapies to overcome cancer immune resistance. In recent years, immune checkpoints blockade therapy has made great progress in the treatment of various cancers (53–55). Especially, agents targeted immune checkpoints such as PD-1, PD-L1 and CTLA-4 have achieved great clinical success in anti-cancer practice (15).

CRISPR/Cas technology can be applied to knock out PD-1 or CTLA-4 in cytotoxic T lymphocytes. Cytotoxic T lymphocytes (CTLs) are the primary immune cells that are responsible for killing cancer cells. The function of CTLs may be attenuated by acquired immune resistance induced by the increased PD-1/CTLA-4 signaling. The blockade of PD-1 or CTLA-4 pathway could enhance the immune response of cancer patients (56). It was reported that the CRISPR/Cas9 technology can be used to effectively knock out PD-1 in CTLs, which could enhance the cytotoxic effect of CTLs against tumor cells (57). Compared with the control, the PD-1 knocked out (KO) CTLs secret more TNF-α and IFN-γ and the xenografted mice treated with PD-1 KO CTLs have longer survival time (58). The CTLA-4 knocked out (KO) CTLs displayed increased cytokine secretion and enhanced anti-tumor activity *in vivo* (58). To sum up, using CRISPR/Cas technology to knock out PD-1 or CTLA-4 in cytotoxic T lymphocytes could enhance the cancer immune response.

Researchers have focused on the study on the application of CRISPR/Cas9 technology on the PD-1 or PD-L1. Those mice inoculated with the murine ovarian cancer cells depleted PD-L1 by CRASPR/Cas9 showed longer survival time than the mice inoculated with control cells (59). Further study showed that the destruction of PD-L1 on tumor cells could increase tumor infiltrating lymphocytes (TILs) and regulate cytokine/chemokine distribution in the tumor microenvironment (TME), thus promoting anti-tumor immunity and inhibiting the ovarian cancer progression (59). Furthermore, study showed that the CTLs cells whose PD-1 was disrupted by CRISPR/Cas9 system have enhanced immune response to the EBV-LMP2A antigen and superior cytotoxicity to the Epstein-Barr virus-positive gastric cancer (60). The result from a human phase I clinical trial revealed that deletion of the genes encoding endogenous T cell receptor (TCR) chains, TCRα (TRAC), TCRβ (TRBC) and programmed cell death protein 1 (PD-1; PDCD1) in T cells resulted in durable engraftment with minimal immunogenicity (61). The CRISPR/Cas9 technology has been established to engineer primary T cells to reduce PD-1 expression (62). It was reported that, in anaplastic lymphoma kinase (ALK)-positive anaplastic large-cell lymphoma (ALK+ ALCL), the CRISPR/Cas9 library was used to identify molecular effectors required for PD-L1 regulation which will provide opportunities for the improvement of immunotherapeutic intervention strategies (63). In conclusion, the application of CRISPR/Cas9 on PD-1 or PD-L1 may provide a direction of cancer immunotherapy.

**Figure 2:**
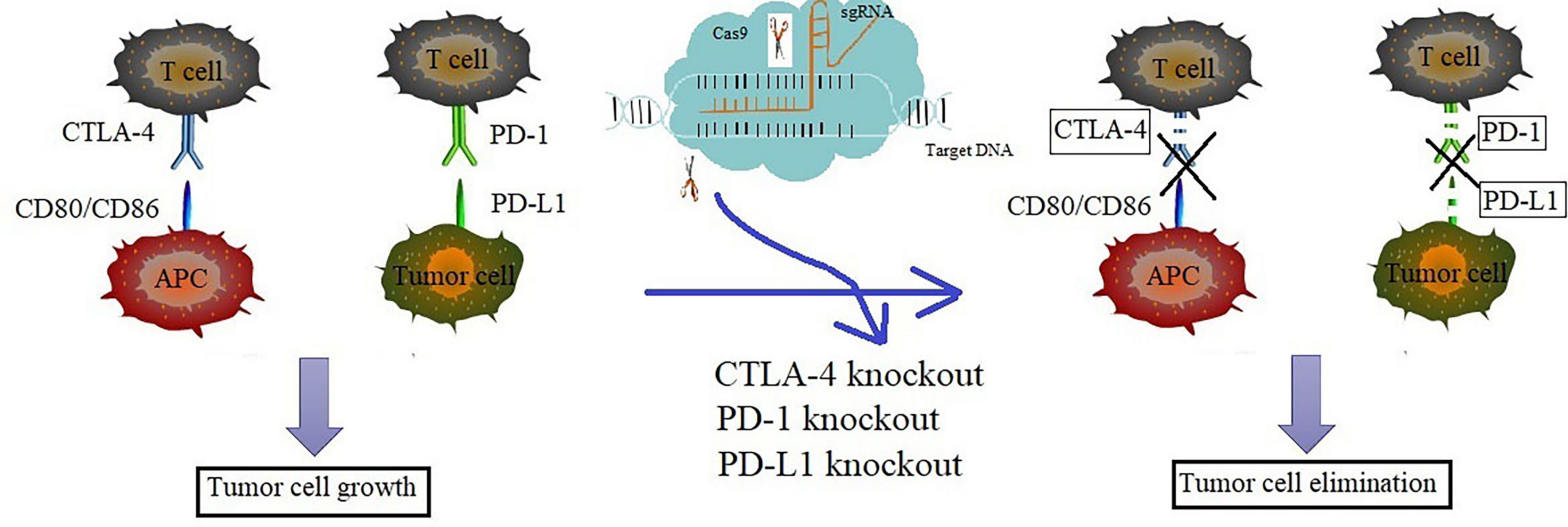
Knockout of PD-1/PD-L1 or CTLA-4 *via* CRISPR/Cas9 enhanced the anti-tumor immune response. The increased level of PD-1 in T cells, PD-L1 in tumor cells and CTLA-4 in APC cells may cause acquired immune resistance and attenuates the function of CTLs. The depletion of PD-1, PD-L1 or CTLA-4 *via* CRISPR/Cas9 technology could overcome the cancer immune resistance and enhance cancer immunotherapy effect. Blue represents the progress of immunotherapy, while pink represents the progress of CRISPR/Cas9 technology. The mixed color represents the key studies of the combined two technologies.

Using CRISPR/Cas9 system to knock out immune checkpoints including PD-1, PD-L1 and CTLA-4 benefits immune checkpoints blockade therapy which may provide a potential strategy to target immune checkpoints. Therefore, the application of CRISPR/Cas9 in immunotherapy will promote the further development of effective immunotherapy in the future (**Figure 2**).

## The Application of CRISPR/Cas9 in CAR-T Cell Therapy

CAR-T cell therapy has shown anti-tumor effect in multiple types of tumors, especially hematological tumors (8). The chimeric antigen receptor (CAR) is a modular fusion protein which consists of a transmembrane domain, a spacer domain, an intracellular signaling domain and a single-chain variable fragment (scFv) specific to an antigen on cancer cells (64). CAR-modified T cells can specifically recognize the tumor-associated antigens (TAA) through the scFv domain, resulting in T cell activation independent of major histocompatibility complex (MHC) restriction (65). Clinical trials have shown that CAR-T cell therapy has promising effect in treating B-cell malignancies, especially in treating acute lymphoblastic leukemia (ALL). For example, the complete remission rate of CD19-specific CAR-T cell therapy in the treatment of ALL is as high as 90% (22).

However, in order to get enough qualified T cells from cancer patients, researchers pay more attention to produce universal or off-the-shelf CAR-T cells from healthy donors. Graft-versus-host disease (GVHD) and biological safety to obtain more powerful disease-targeted activity are two main obstacles in producing universal CAR-T cells (66). The endogenous αβ T-cell receptor (TCR) on allogeneic CAR-T cells may recognize recipient alloantigens and cause GVHD. The human leukocyte antigen (HLA) molecules on allogeneic T cells are recognized as foreign HLA molecules and lead to immunologic rejection in recipients.

The CRISPR/Cas9 technology has been used to produce CAR-T cells with disrupted TCR beta chain and beta-2-microglobulin (B2M), an essential subunit of the HLA-I (16, 17). These modified CAR-T cells can retain the desired antitumor function without causing GVHD (16, 17). Therefore, CRISPR/Cas9 technology provides a direction to produce universal or off-the-shelf CAR-T cells, which is of great importance to generate more and cheaper CAR-T cells.

**Figure 3:**
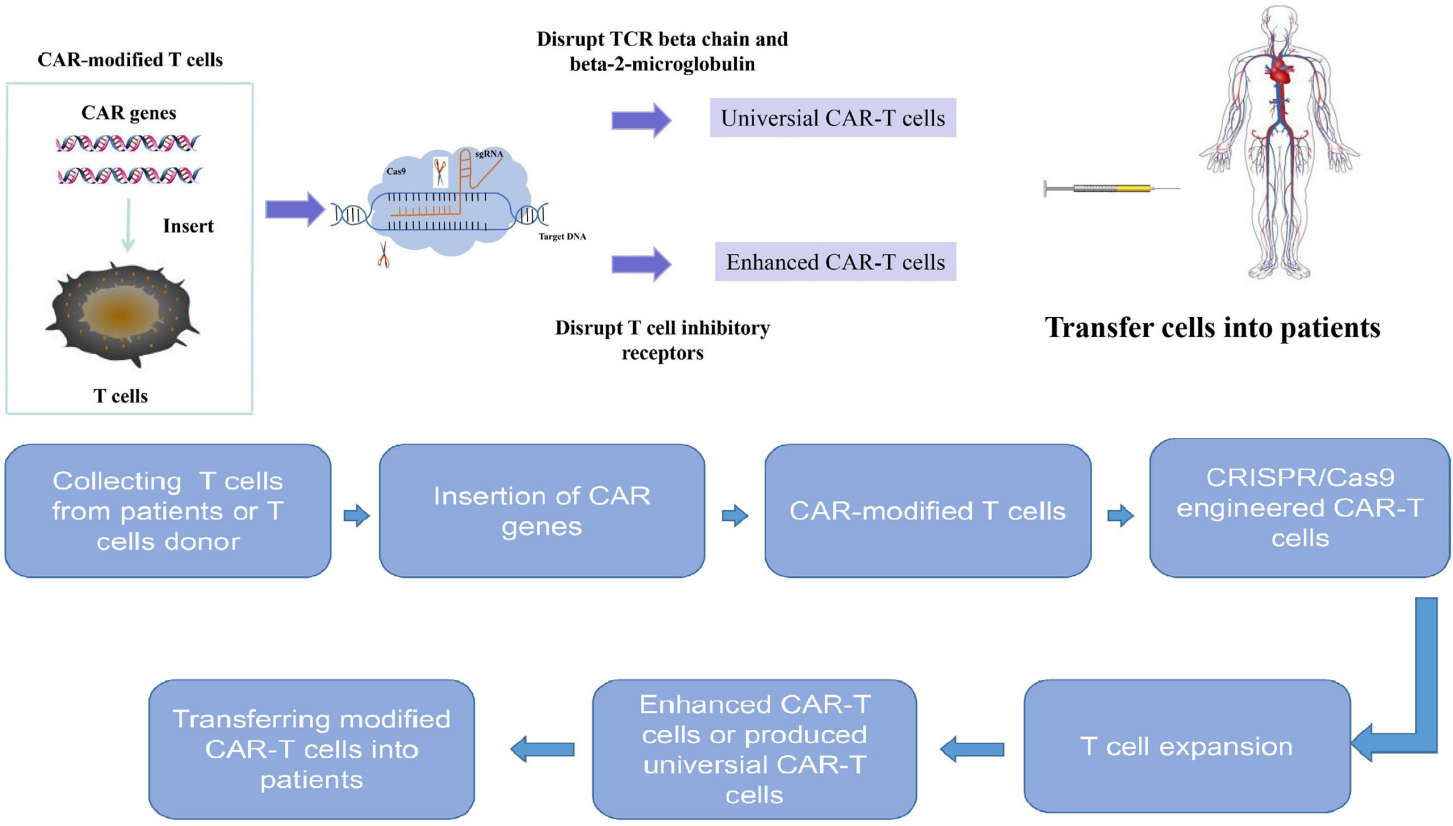
The treatment process for combination of CRISPR/Cas9 technology and chimeric antigen receptor T cell therapy. CRISPR/Cas9 system can be used to genentate universal CAR-T cells and enhance CAR-T cell efficacy. The treatment progress is as follows: T cells are collected from peripheral blood of patients and further activated and expanded. Then the chimeric antigen receptor genes were inserted into T cells to generate chimeric antigen receptor modified T cells. The chimeric antigen receptor T cells are engineered by CRISPR/Cas9 system to generate universal chimeric antigen receptor T cells or enhanced chimeric antigen receptor T cells. Then these engineered chimeric antigen receptor T cells are expanded *in vitro* and later transferred back to patients.

Moreover, CRISPR/Cas9 technology can also be used to enhance the anti-tumor effect of CAR-T cells. Double-knockout (TRAC and B2M) and triple-knockout (TRAC, B2M and PD-1) CAR-T cells *via* CRISPR/Cas9 technology showed superior anti-tumor activity (67). Moreover, CRISPR/Cas9 technology has also been reported to improve the anti-tumor effect and clinical outcome of CAR-T cells by disrupting T cell inhibitory receptors, such as PD-1 (68) and Lymphocyte activation gene-3 (LAG3) (69). It was also reported that the PD-1 depleted CAR-T cells by CRISPR/Cas9 system displayed enhanced anti-tumor efficacy against hepatocellular carcinoma (HCC) (70). In addition, using CRISPR/Cas9 to knock out diacylglycerol kinase (DGK) in CAR-T cells can also potentiate the effector functions of CAR-T cells *in vitro* (71). Universal CAR-T cells resistant to PD-1 inhibition have been created by disrupting endogenous T-cell receptor (TRAC), beta-2-microglobulin (B2M) and PD-1 *via* CRISPR/Cas9 gene-editing system, and these triple gene-edited CAR-T cells showed enhanced antitumor activity in mice bearing intracranial tumors (72). The TGF-β receptor II (TGFBR2) depleted CAR-T cells by CRISPR/cas9 promoted tumor elimination efficacy of CAR-T cells both *in vivo* and *in vitro*, which provides a novel method to improve CAR-T cells’ function in the TGF-β-rich tumor environment and promote CAR-T cells’ efficacy in solid tumors (73). Compared to wild type CAR-T cells, the granulocyte macrophage colony-stimulating factor (GM-CSF) depleted CAR-T cells by CRISPR/Cas9 produced less GM-CSF and resulted in better anti-tumor activity *in vivo* (74). Eyquem et al. reported that directing a CD19-specific CAR into the T-cell receptor α constant (TRAC) locus by electroporation of Cas9 mRNA and sgRNA not only results in uniform CAR expression, but also enhances T-cell potency in an acute lymphoblastic leukaemia (ALL) mouse model (75). Fas receptor which is involved in T cell response is a member of TNF family (76). The Fas receptor and its ligand (FasL) participate in T cell apoptosis and attenuate CAR-T cell activity (77). It has been reported that CAR-T cell activity is attenuated by Fas-FasL dependent activation induced cell death (AICD) (78). The CAR-T cells depleted of Fas receptor by CRISPR/Cas9 showed resistance to apoptosis and enhanced anti-tumor efficiency (79). In a recent study, Cbl-b has been identified as a potential target to overcome CAR-T cell exhaustion based on RNA-sequencing data from CD8+ tumor infiltrating lymphocytes (TILs) (80). The deletion of Cbl-b in CAR T cells makes them resistant to exhaustion (80). Recently, Olli Dufva et al. used more than 500 small-molecule drugs and CRISPR/Cas9 screening to identify key pathways of CAR-T cell toxicity, paving the way for avoiding CAR T cell cytotoxicity (81). Dongrui Wang et al. identified the essential factors influencing CAR-mediated glioblastoma killing based on CRISPR screens in CAR T cells and patient-derived GBM stem cells (82). Therefore, CRISPR/Cas9 could help to optimize the manufacture of CAR-T cells by silencing or disrupting desired genomic loci and improve therapeutic effect of CAR-T cell therapy (**Figure 3**).

## The Application of CRISPR/Cas9 in CAR-NK Cell Therapy

Natural killer (NK) cells can kill cancer cells directly without recognizing tumor-specific antigen (83). Compared to CAR-T cell therapy, CAR-NK cell therapy has advantages on safety. Cytokine release syndrome (CRS) is one of the most severe toxicities of CAR-T cell therapy due to proinflammatory cytokines including tumor necrosis factor α (TNFα), interleukin-1 (IL-1) and interleukin-6 (IL-6) produced by the activation and proliferation of CAR-T cells (84). The CAR-NK cell therapy reduced possibility of CRS as the cytokines produced by NK cells are mainly composed of Interferon-γ (IFN-γ) and granulocyte-macrophage colony-stimulating factor (GM-CSF). CAR-NK cells transferred into tumor patients will not expand within a few weeks. Studies also showed that NK cells do not cause GVHD in the allogeneic setting, providing opportunities to produce off-the-shelf cellular therapy products (85).

CRISPR/Cas9 has been applied in CAR-NK cell therapy to enhance the anti-tumor activity of NK cells. May Daher et al. used CRISPR/Cas9 technology to delete CISH in CAR-NK cells and the modified CAR-NK cells have better metabolic fitness and antitumor activity (86). Mark Gurney et al. used CRISPR/Cas9 technology to disrupt the *CD38* gene during expansion with a mean knockdown efficiency of 84% to achieve an affinity optimized CD38 CAR (87). The CD38 knockout NK cells expressing an affinity optimized CD38 CAR showed reduced NK cell fratricide and enhanced ability to target acute myeloid leukemia (AML) (87).

## The Application of CRISPR/Cas9 in Macrophage-Based Therapy

Immune escape plays an important role in tumor growth and development. One of the mechanisms of immune escape is the “don’t eat me” signals generated from tumor cells to prevent macrophage mediated phagocytosis (88). CD47, a cell surface protein which is overexpressed on most cancer cells, is an important “don’t eat me” signal (89). Even at the present of phagocytic signal, the interaction between CD47 and macrophage signal regulatory protein-α (SIRP-α) could bypass phagocytosis (90). The underlying mechanism of CD47:SIRP-α binding mediated phagocytosis inhibition is that their binding leads to activation of SIRP-α by phosphorylation of its receptor (91). Subsequently, the phosphorylation results in the binding and activation of homologous phosphatase-1 (SHP-1) and SHP-2 (92), thus inhibiting the phagocytosis by preventing the accumulation of myosin-IIA at phagocytic synapse. Therefore, strategies should be developed to avoid the immune escape.

The CRISPR/Cas9 technology can be used to avoid the “don’t eat me” signals and enhance the function of macrophages. Before the application of CRISPR/Cas9 technology on macrophages, strategies to block the binding of CD47 and SIRP-α to turn off the “don’t eat me” signals have been developed, such as application of anti-CD47 monoclonal antibody (93) and the engineered SIRP-α variant adjuvant consensus variant 1 (CV1) (94). Although these strategies have shown high efficacy, the antigen sink caused by overexpressed CD47 on cancer cells reduces bioavailability and increases potential toxicity to normal cells (95). Interestingly, in one study, CRISPR/Cas9 system has been applied to knock out SIRP-α in macrophages and subsequently enhanced the ability of macrophages to phagocytose cancer cells which providing a new immunotherapeutic method for cancer therapy (18). Altogether, using CRISPR/Cas9 system to knock out specific genes in macrophages can minimize the impact of suppressive tumor microenvironment and improve antitumor immune response, which may be of great significance for the development of tumor therapy in the future.

## Safety Problems

Although CRISPR/Cas9 technology has provided a convenient and efficient strategy to help to optimize tumor immunotherapy, there are still some potential safety risks. One of the serious problems of CRISPR/Cas9 technology is the off-target, because unexpected off-target mutagenesis may cause the ablation of tumor-suppressor genes or the activation of oncogenes (96). Recent studies have reported the off-target effects of CRISPR/Cas9 technology in editing T cells (97). Furthermore, studies also indicated that CRISPR/Cas9 technology may inadvertently cause cancer (96). It has been found that Cas9 RNP delivery induces p53-mediated DNA damage response in human retinal pigment epithelial cells (97). The activation of p53 may cause chromosome rearrangement and other oncogenic mutations. Moreover, studies have shown that CRISPR RNAs may trigger innate immune responses in cells, leading to cytotoxicity (98).

Another safety issue is that the multiple gene editing may cause the translocations between double-strand break sites. Translocation frequencies ranged from 10^-4^ to 2×10^-2^ have been reported in T cells treated with CD52 and TRAC TALENs (99). Although there was no proliferation advantage detected in translocated T cells, thorough transformation analysis is required to confirm the safety of multiple gene edited therapy. Clinical safety of the application of CRISPR/Cas9 technology in immunotherapy is also an important concern. Genetically engineered viruses may cause unexpected toxic reactions (100). The off-target issues of CRISPR/Cas9 technology may increase the clinical risks of therapy. Therefore, it warrants further in-deep investigation on the possible mechanisms that might influence the safety of the combination of CRISPR/Cas9 technology and immunotherapy.

## Discussion

The development of immunotherapy is of great significance in the cancer treatment history. CRISPR/Cas9 technology may further optimize immunotherapy, improve the anti-tumor effect and expand the application scope of immunotherapy by targeting special genes. However, there are many issues that should be considered in the application of CRISPR/Cas9 system in immunotherapy, such as off-target effect, editing efficiency and clinical safety which provide challenges to researchers.

Researchers have explored many approaches to reduce the off-target effects and improve the specificity of CRISPR/Cas9 technology. The most effective way to improve the specificity is to select the appropriate target sequence. Studies indicated that the off-target mutagenesis mediated by CRISPR/Cas9 may be different due to different sgRNA design and target sequences (101). The development of predictive algorithms provides the strategy to reduce the off-target effects by computationally searching target sequences that bear the least similarities to other sequences. Studies have shown the precise regulation of the number of Cas9 and sgRNA also helps to improve specificity of CRISPR/Cas9 technology. The off-target effects can be reduced by reducing CRISPR reagents in cells (102). Compared with plasmids and viruses, editing with cas9 mRNA and protein can reduce the off-target effects because of the rapid degradation of mRNA or RNPs after cleavage on the target (98). The modification of sgRNA sequence can also reduce the off-target effects. Studies showed that sgRNA with truncated base-pairing sequences (17 nt instead of 20 nt) can improve the targeting specificity, which may be because truncated sgRNA reduced binding affinity to the target DNA and thus improve the sensitivity to mismatches (102). Cas9 nickase, which contains mutations in the nuclease domains HNH or RuvC, is an alternative approach to improve the specificity in editing (103). Fusing catalytically inactive Cas9 to FokI nuclease can also enhance the CRISPR specificity and reduce the unwanted mutagenesis, but the efficiency of gene editing may be reduced (104). Meanwhile, some other CRISPR/Cas systems were also developed to edit the genome with higher efficiency and target specificity. Given that CRISPR/Cas9 is the firstly developed genomic editing method, however, we mainly focus on CRISPR/Cas9 system in this review.

The editing efficiency also affects the application of CRISPR/Cas9 technology. Researchers improve the editing efficiency mainly through the optimization of the internal sequence in CRISPR/Cas9 gene editing system, the improvement of gene editing delivery system and the optimization of gene editing repair strategy (105).

Slaymaker et al. hypothesized the positively-charged residues are related to the stability of the non-target DNA strand and produced systematic single or multiple mutations in these residues (106). Then Cas9 mutants which could improve the precision of genomic editing without impairing on-target activity are identified in this study (106). Kleinserver et al. developed an amino acid substituted SpCas9 with exceptional precision using the similar approach (107). However, high-fidelity Cas9 variants need further exploration to improve the reliability of CRISPR/Cas9 system as a tool for cancer therapy.

Although novel strategies have been developed to improve the specificity of CRISPR/Cas9 gene editing and reduce off-target frequency, the degree of accuracy of gene editing remains to be determined in clinical practice. In addition, how the autoimmune system will respond to genetically engineered cells is still not fully studied. Excitingly, the encouraging results of universal CAR-T therapy in research and clinical application indicated that CRISPR/Cas9 technology showed a promising future in comprehensive tumor treatment based on tumor immunotherapy (108). Therefore, although there are some problems and challenges in the application of combination of CRISPR/Cas9 and immunotherapy, the continuous progress of CRISPR/Cas9 technology will contribute more to cancer immunotherapy in the near future.

## Author Contributions

LW wrote the manuscript. LW and YC drew the pictures. ZL and XD revised the manuscript. XD reviewed and edited the manuscript. All authors contributed to the article and approved the submitted version.

## Funding

This work was partly supported by the National Natural Science Foundation of China (No: 81972558), the “Startup funding of First Hospital, JLU”, the Natural Science Foundation of Jilin (No: 20200201367JC).

## Conflict of Interest

The authors declare that the research was conducted in the absence of any commercial or financial relationships that could be construed as a potential conflict of interest.

## Publisher’s Note

All claims expressed in this article are solely those of the authors and do not necessarily represent those of their affiliated organizations, or those of the publisher, the editors and the reviewers. Any product that may be evaluated in this article, or claim that may be made by its manufacturer, is not guaranteed or endorsed by the publisher.
