# Supplementary material for: The Application of CRISPR/Cas9 Technology for Cancer Immunotherapy: Current Status and Problems
Source: Front Oncol. 2022 Jan 17;11:704999. doi: 10.3389/fonc.2021.704999 (PMC8801488; doi:10.3389/fonc.2021.704999)
Supplement: Supplementary file 1 [file Table_1.doc]

Table 1 Summarization of the application of CRISPR/Cas9 in tumor immunotherapy.

| **The application of the CRISPR/Cas9 technology in tumor immunotherapy** | **The results of application of the CRISPR/Cas9 technology** | Ref. |
| --- | --- | --- |
| CRISPR-based high-throughput screening | Identifying the essential genes in tumor cells for the EFT | 39 |
| CRISPR screening | Figuring out FAM49B as a novel target of tumor immune drugs to improve the anti-tumor ability of T-cells | 42 |
| Genome-wide CRISPR-Cas9 screen | Exploring the mechanism underlying immunomodulatory drug IMiDs sensitivity | 41 |
| Introducing DsRed gene into ADV genome | Creating mutations in large viral DNA genomes | 47, 48, 49 |
| Knocking out PD-1 in CTLs | Enhancing the anti-tumor efficiency of CTLs | 57 |
| Knocking out CTLA-4 in CTLs | Increasing cytokine secretion and enhancing anti-tumor efficiency | 58 |
| Depleting PD-L1 in murine ovarian cancer cells | Prolonging survival times of mice | 59 |
| Disrupting PD-1 in the CTLs cells | Enhancing cytotoxicity to the EBV positive gastric cancer | 60 |
| Deleting the genes encoding endogenous TCR chains, TRAC, TRBC and PD-1 in T cells | Durable engraftment of engineered T cells | 61 |
| Using CRISPR/Cas9 library to identify molecular effectors required for PD-L1 regulation in ALK+ ALCL | Providing opportunities for the improvement of immunotherapeutic intervention strategies | 62 |
| Disrupting TCR beta chain and B2M in CAR-T cells | Producing universal CAR-T cells | 11, 12 |
| Cas9:single-guide RNA ribonucleoproteins technology | Engineering primary T cells to reduce PD-1 expression | 63 |
| Double-knockout (TRAC and B2M) and triple-knockout (TRAC, B2M, and PD-1) CAR-T cells | Producing universal CAR-T cells | 68 |
| Disrupting PD-1 in CAR-T cells | Improving the anti-tumor efficiency and clinical outcome | 69, 71 |
| Disrupting LAG3 in CAR-T cells | Improving the anti-tumor efficiency and clinical outcome | 70 |
| Knocking out DGK in CAR-T cells | Potentiating the effector functions | 72 |
| Disrupting endogenous TRAC, B2M and PD-1 in CAR-T cells | Producing universal CAR-T cells resistant to PD-1 inhibition | 73 |
| Depleting TGFBR2 in CAR-T cells | Promoting the tumor elimination efficacy | 74 |
| Depleting GM-CSF in CAR-T cells | Producing less GM-CSF and resulting in better anti-tumor efficiency | 75 |
| Directing a CD19-specific CAR into the TRAC locus by electroporation of Cas9 mRNA and sgRNA | Achieving uniform CAR expression and enhancing T-cell potency in a mouse model of ALL | 76 |
| Depleting Fas receptor in CAR-T cells | Enhancing anti-tumor efficiency | 80 |
| Knocking out SIRP-α in macrophages | Enhancing the anti-tumor efficiency of macrophages | 13 |
| Deleting Cbl-b in CAR-T cells | Making CAR-T cells resistant to exhaustion | 82 |
| CRISPR screening | Identifying key pathways of CAR-T cell toxicity | 83 |
| CRISPR screening | Identifying essential factors influencing CAR-mediated glioblastoma killing | 84 |
| Deleting CISH in CAR-NK cells | Enhancing metabolic fitness and antitumor activity of CAR-NK cells | 88 |
| Disrupting the CD38 gene during expansion | Reducing NK cell fratricide and enhancing antitumor ability | 89 |
