# Supplementary material for: The Application of CRISPR/Cas9 Technology for Cancer Immunotherapy: Current Status and Problems
Source: Front Oncol. 2022 Jan 17;11:704999. doi: 10.3389/fonc.2021.704999 (PMC8801488; doi:10.3389/fonc.2021.704999)
Supplement: Supplementary file 2 [file Table_2.doc]

Table 2 Summarization of abbreviations and their full name.

| **Abbreviation** | **Full name** |
| --- | --- |
| CAR-T | Chimeric antigen receptor T |
| CRISPR-Cas9 | Clustered regularly interspaced short palindromic repeats-associated 9 |
| TCR | T-cell receptor |
| HLA | Human Leukocyte Antigen |
| B2M | Beta-2-microglobulin |
| SIRP-α | Signal regulatory protein-α |
| sgRNA | single guide RNA |
| ZFNs | Zinc-finger nucleases |
| TALENs | Transcription activator-like effector nucleases |
| CTLA-4 | Cytotoxic T-lymphocyte antigen 4 |
| PD-1 | Programmed death-1 |
| JAK1 | Janus kinases 1 |
| JAK2 | Janus kinase 2 |
| EFT | Effector function of T cells |
| 2CT | Two cell-type |
| OVs | Oncolytic viruses |
| T-VEC | Talimogene laherparepvec |
| GM-CSF | Granulocyte-macrophage colony-stimulating factor |
| BAC | Bacterial artificial chromosome |
| VV | Vaccinia virus |
| HSV | Herpes simplex virus |
| ADV | Adenoviral vector |
| kDa | Kilodalton |
| TK | Thymidine kinase |
| CTLs | Cytotoxic T lymphocytes |
| TILs | Tumor infiltrating lymphocytes |
| TME | Tumor microenvironment |
| scFv | Single-chain variable fragment |
| TAA | Tumor-associated antigen |
| MHC | Major histocompatibility complex |
| ALL | Acute lymphoblastic leukemia |
| GVHD | Graft-versus-host disease |
| HCC | Hepatocellular carcinoma |
| DGK | Diacylglycerol kinase |
| TGFBR2 | TGF-β receptor II |
| AICD | Activation induced cell death |
| CRS | Cytokine release syndrome |
| IL-1 | Interleukin-1 |
| IL-6 | Interleukin-6 |
| AML | Acute myeloid leukemia |
| SIRP-α | Signal regulatory protein-α |
| IMiDs | Immunomodulatory drugs |
| EBV | Epstein-Barr virus |
| LMP2A | Latent membrane protein 2A |
| ALK+ ALCL | Anaplastic lymphoma kinase-positive anaplastic large-cell lymphoma |
| LAG3 | Lymphocyte activation gene-3 |
| TRAC | T-cell receptor α constant |
| TNFα | Tumor necrosis factor α |
| IFN-γ | Interferon-γ |
| RNPs | RGEN ribonucleoproteins |
| CV1 | Consensus variant 1 |
